# Comparing the efficacy of duloxetine and nortriptyline in alleviating the symptoms of functional dyspepsia – a randomized clinical trial

**DOI:** 10.3389/fpsyt.2023.1297231

**Published:** 2024-01-16

**Authors:** Seyed Shahab Banihashem, Seyedeh Mahsa Mofatioshieh, Reyhaneh Rastegar, Amir Sadeghi

**Affiliations:** ^1^Department of Psychosomatic Medicine, Taleghani Hospital, School of Medicine, Shahid Beheshti University of Medical Sciences, Tehran, Iran; ^2^School of Medicine, Shahid Beheshti University of Medical Sciences, Tehran, Iran; ^3^Gastroenterology and Liver Disease Research Center, Research Institute for Gastroenterology and Liver Disease, Shahid Beheshti University of Medical Science, Tehran, Iran

**Keywords:** functional gastrointestinal disorders, antidepressant, functional dyspepsia, Nortriptyline, Duloxetine

## Abstract

**Aim:**

To compare the efficacy of Duloxetine and Nortriptyline in alleviating the symptoms of severity, anxiety, depression and quality of life in patients with functional dyspepsia (FD).

**Material and method:**

We conducted a single-blinded 3-month trial of Duloxetine 20–30 mg daily in 20 patients and Nortriptyline 25 mg daily in 25 FD patients. The primary outcome measure was the severity of FD symptoms by Gastrointestinal symptoms rating scale. Secondary measures included Hamilton Anxiety Rating Scale, Hamilton Depression Rating Scale, and Nepean Dyspepsia Index. the patients were measured in 3 stages.

**Results:**

45 patients with FD with a mean age of 37.18 ± 10.62 years participated in the study. The severity of symptoms was significantly lower in the Nortriptyline group than in the Duloxetine group after three months (*p* = 0.031). The level of anxiety (*p* = 0.049), depression (*p* = 0.045) and quality of life (*p* = 0.046) improved significantly after three months in the Duloxetine group compared to Nortriptyline. Mediation analysis using linear regression revealed a significant mediator role for anxiety. This mediation analysis revealed a 21.13% reduction in anxiety in the Duloxetine group.

**Conclusion:**

While both medications demonstrated efficacy, Nortriptyline appeared to be superior in symptom reduction. Duloxetine exhibited more advantages compared to Nortriptyline in addressing anxiety and depression and enhancing the overall quality of life. Also, Duloxetine may have a noteworthy impact, contributing to a 20% reduction in FD symptoms by lowering anxiety levels.

**Clinical trial registration:**

https://en.irct.ir/trial/65512.

## Introduction

Functional dyspepsia (FD) is a prevalent functional gastrointestinal disorder characterized by symptoms such as epigastric pain, burning, postprandial fullness, and early satiety. Experience of these symptoms for patients is irritating. Unlike other gastrointestinal conditions, FD lacks evidence of organic abnormalities, making it a challenging diagnosis. Symptoms must persist for at least three months, with an onset of at least six months before diagnosis (1). The prevalence of FD is significant, affecting approximately 21.8% of the population (2).

The pathophysiology of FD involves a complex interplay of factors, including visceral hypersensitivity, impaired gastric accommodation, hypersensitivity to luminal contents, small bowel dysmotility, psychological disturbances, central nervous system disorders, and *Helicobacter pylori* infection (3, 4). Notably, a holistic biological-psycho-social model suggests that psychological issues like anxiety and depression may disrupt sensory filtering, increasing physiological sensory signals in visceral areas, thereby impacting gastrointestinal function and disrupting it (5–8).

A prominent perspective on FD’s pathophysiology emphasizes disruptions in the brain-gut axis (9, 10). The brain and the gastrointestinal system play vital roles as sensory organs, actively detecting, transmitting, combining, and reacting to signals originating from both inside and outside the body. At the point where these sensory functions converge, immune cells in the intestines and the brain continually monitor environmental factors, triggering responses that provide information about the body’s overall physiological condition (11). The disturbance in this axis lead to changes in gut motility and regional transit, visceral sensitivity, immune function, and mood (12).

Understanding the connection between the brain-gut axis and the role of psychiatric medicine is critical. The enteric nervous system, originating from embryonic endoderm and influenced by neural cells from the brain and spinal cord during fetal development, shares neurotransmitters and receptors with the central nervous system. This shared communication system plays pivotal roles in both brain and gut function. Importantly, since many antidepressants affect these common neurotransmitters and receptors, they can influence gastrointestinal symptoms (13).

Several classes of antidepressant pharmacological treatment such as tricyclic antidepressants (TCAs) selective serotonin reuptake inhibitors (SSRIs), and serotonin and norepinephrine reuptake inhibitors (SNRI) have demonstrated benefits in FD (14). Current guidelines recommend these antidepressants as a second-line drug especially after failure of PPI and/or prokinetics (4, 13). However, the efficacy of these drugs for the treatment of FD is controversial (13), and choosing the most appropriate drug therapy in terms of safety and tolerability is difficult. In addition, due to the increasing prevalence of FD, pharmacological treatment options remain limited. Therefore, developing new pharmacological options to treat this disorder is a priority (3, 4).

We reasoned that the antidepressant Duloxetine, a SNRI, already shown efficacious in other pain syndromes might represent a promising alternative to SSRIs and TCAs and 5-hydroxytryptamine (5-HT)-1A receptor agonists for FD. To investigate this possibility, we conducted a single-blinded clinical trial comparing Duloxetine’s effects with Nortriptyline, a TCA, in patients with FD. This study marks the first exploration of Duloxetine’s efficacy in FD patients The reason we chose SNRI is that there have not been enough trials to investigate the effect of SNRs on chronic gastrointestinal pain (15) and on the other hand has fewer side effects than TCA (16), so the results of this research can help future treatment policies. Among the SNRIs, we chose Duloxetine because it has clinically significant noradrenergic effects even in the low dose range at the start of treatment. Also they do not have antihistaminic or anticholinergic side effects (16).

## Patients and method

### Study design

The present study is a randomized clinical trial, single-blinded with paralleled group. The study protocol approved by ethics committee of Shahid Beheshti University of medical sciences (ID: IR.SBMU.MSP.REC.1400.176). Also, it is approved in registry of clinical trial center (code: IRCT20220329054367N2).

### Study size

The determination of the sample size was an intricate process that involved multiple considerations. Rather than relying on a specific previous study, insights were drawn from a pilot study, and existing literature in the field of functional dyspepsia was extensively reviewed. This comprehensive approach allowed for the consideration of variability in outcome measures, effect sizes, and relevant parameters from studies with similar interventions and patient populations.

The primary outcome guiding the sample size determination was the change in the severity of functional dyspepsia symptoms, a crucial measure for evaluating the effectiveness of the interventions under investigation. To ensure robust statistical power across all relevant endpoints, a careful approach was adopted by considering the most conservative outcome among dimensions assessed, including GSRS (dyspepsia, abdominal pain, and nausea), Hamilton Anxiety Rating Scale, Hamilton Depression Rating Scale, and Nepean Dyspepsia Index.

The sample size calculation employed a standard two-sample *t*-test formula:


n=2.Z1−α2+Z1−β2.σ2δ2

For our specific calculation, we selected the following parameters of *α* = 0.05, 1-β = 0.8, *σ* = 6, and *δ* = 5.

Plugging these values into the formula, we arrived at a required sample size of 22 per group.

### Participants

From September 2022 to March 2023, patients aged 18–60 years diagnosed with FD according to ROME-IV criteria were invited to participate in this study. Inclusion criteria considered for this study included normal upper endoscopy with no organic symptoms, and failure to respond to Proton Pump Inhibitors (PPI) treatment. Exclusion criteria were defined as follows: individuals with comorbidities associated with other gastrointestinal disorders, a history of drug or alcohol abuse, underlying psychiatric disorders, or those who had been taking psychotropic drugs within the past six months. Additionally, individuals with known allergies to psychotherapeutic drugs, those who did not adhere to prescribed medication regimens, those who withdrew their participation in the project, and pregnant women were excluded from the study. Before commencing the intervention, a diagnostic interview was conducted by a psychiatrist to rule out the presence of mental disorders in the participating individuals.

**Figure 1:**
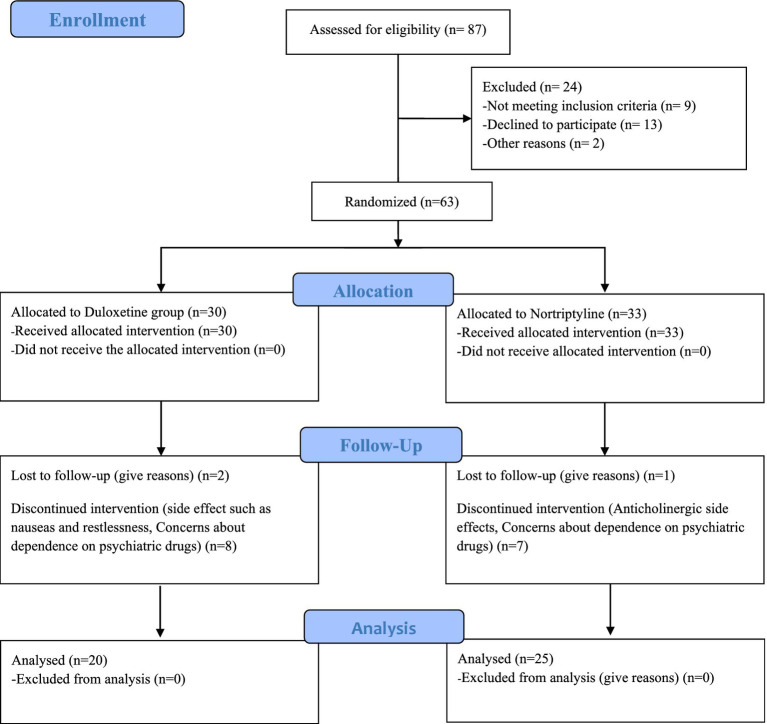
CONSORT flow diagram.

63 patients were included in the study. During the intervention, 18 patients, including 10 in the Duloxetine group and 8 in the Nortriptyline group, were excluded from the study due to unwillingness to continue participating in the study or drug intolerance. Finally, 45 people remained until the end of the treatment. The CONSORT flow diagram (Figure 1) show the phases of this parallel randomized trial of two groups in more details.

### Randomization and intervention

Appreciation is extended for expressing interest in the random assignment approach employed in the study. In the randomized clinical trial, transparency and unbiased allocation of participants to treatment arms were prioritized. After the initial diagnosis of functional dyspepsia by a gastroenterologist, eligible patients underwent further evaluation by a psychiatrist. To ensure unbiased allocation, a block randomization method with a block size of four, accommodating three patients per block in accordance with the order of enrollment, was utilized.

The method aimed to equally distribute potential confounding factors between treatment groups. The online block randomization software ‘Sealed Envelope’ was employed to execute the randomization process, generating randomized block lists enclosed in sealed envelopes. Each day, a new envelope was opened by the psychiatrist to determine patient allocation, ensuring a concealed and unbiased assignment.

Patients were randomly assigned to the Duloxetine arm or the alternative treatment arm with Nortriptyline, with careful consideration to minimize selection bias. The study maintained a single-blinded design, preventing participants from knowing their assigned treatment to minimize bias in outcome assessment. This blinding strategy was consistently applied throughout the study period, including baseline, 1 month, and 3 months after starting interventions. This rigorous randomization process aimed to distribute potential confounding factors equally between the two treatment groups, enhancing the internal validity and reliability of the study results.

In the Duloxetine arm, patients were administered tablets containing 20 mg of Duloxetine (manufactured by Abidi company) during the first month, followed by an increased dosage of 30 mg in the subsequent two months, to be taken postprandially. In the alternative treatment arm, patients received a daily postprandial dosage of 25 mg of Nortriptyline (manufactured by Abidi company). In both groups, patients also received PPIs as part of their treatment regimen. Patients were advised against the use of over-the-counter medications. After the efficacy in FD symptoms the patients seeking relief from anxiety and depression symptoms chose to undergo continued drug treatment, supervised by the psychiatrist, to address and improve their mental health conditions.

### Outcome assessment

Before intervention baseline characteristics including age, gender and functional dyspepsia symptoms were recorded. The patients were assessed by four questionnaires:

*Gastrointestinal symptoms rating scale (GSRS):* This 15-question questionnaire evaluates the severity of digestive symptoms according to a seven-point Likert scale from no discomfort (0) to severe discomfort (7). This tool has 5 dimensions including heartache, reflux, diarrhea, constipation, and dyspepsia. The total score is obtained from the sum of the scores in each subscale. An increase in the score indicates the severity of the symptoms (17). Mazaheri et al. determined factor structure of GSRS in Persian. Based on their results this scale can be used separately without losing reliability (Cronbach’s alpha = 0.80). In addition, face validity, construct validity, and content validity were acceptable (18). So due to our purpose we used three dimensions including dyspepsia, abdominal pain and nausea.

*Hamilton anxiety rating scale*: This 14 -question tool is used to measure the severity of symptoms of anxiety. Each question has 5 points, which are scored from 0 to 4. The total score is calculated by summing the ratings for all items, with higher scores indicating greater anxiety severity. This scale evaluates some psychological symptoms including nervousness, tension, worry, fears, insomnia, muscle aches, dizziness and sensation, demonstrated high reliability (interrater reliability = 0.74) for precise assessments. Furthermore, internal validity tested by latent structure analysis was insufficient (19).

*Hamilton depression rating scale*: The Hamilton Depression Rating Scale (HDRS) (the 17-item version) assesses various aspects of depression, including mood, guilt, anxiety, sleep disturbances, and physical symptoms. Each item on the scale is scored on a scale from 0 to 4, with higher scores indicating more severe symptoms. The total score is calculated by summing the ratings for all items. Demonstrated high reliability (interrater reliability = 0.74) for precise assessments. Furthermore, internal validity tested by latent structure analysis was insufficient (20, 21).

*The Nepean Dyspepsia Index* (22): NDI is a self-administered questionnaire designed to assess the impact of dyspepsia, a common gastrointestinal disorder characterized by chronic or recurrent discomfort or pain in the upper abdomen. The Nepean Questionnaire is particularly focused on evaluating different aspects of the health-related quality of life of individuals who experience FD symptoms. These aspects can include pain, discomfort, eating habits, social activities, and emotional well-being. Respondents are asked to rate the frequency and severity of their symptoms and the impact on their daily life. This scale has 10 question rating 1 to 5. Higher scores indicate a greater negative impact on quality of life. Validity and reliability assessments were conducted on the NDI. The symptom checklist exhibited good discriminant validity, consistently showing positive scores four times greater than negative scores. Internal consistency of NDI domains was strong, unaffected by weighting. Discriminant validity was confirmed, indicating worse scores for dyspeptic patients. Convergent validity showed moderate correlations between SF-36 and NDI subscales. Dyspepsia subgroups demonstrated consistent NDI performance. The correlation between changes in symptom severity and quality of life scores was hypothesized and indicated a strong, negative association. These results affirm the NDI’s robustness in assessing dyspepsia-related symptoms and their impact on quality of life (23).

The assessment was performed at baseline, 1 months and 3 months after starting interventions. The primary outcome was change in severity of FD symptoms and the secondary outcome was improvement in the level of quality of life, anxiety and depression. Also, side effects of drugs were evaluated.

### Statistical analysis

All statistical analyses in this study were conducted using SPSS version 26 software. Quantitative data were presented as means and standard deviations or median and interquartile ranges, while qualitative data were expressed as counts and percentages. To assess the normality of data distribution, the Shapiro–Wilk test was employed. To compare quantitative data in each group at each time point the Independent Sample *T*-test was utilized for normally distributed data, while the Mann–Whitney *U* test was employed for data with a non-normal distribution. Pearson Chi-square test was used to compare qualitative data at each time point. The generalized estimation equation was used to evaluate the impact of Group [Nortriptyline vs. Duloxetine] on the GSRS, HAS, HDS, and SD-NDI over time, considering Time and Group effect. To examine temporal changes of the quantitively measured data over the time of the study within each group, we employed Repeated Measures ANOVA and non-parametric ANOVA (Friedman’s test) for data with normal and non-normal distributions, respectively. To compare findings within groups at different time points, we utilized the Paired T-test in each group. For the evaluation of mediation effects, we employed path analysis with the PROCESS macro plugin and implemented the Bootstrapping technique. A value of *p* of <0.05 was considered statistically significant.

## Results

### Demographic and characteristics results

A total of 45 patients with functional dyspepsia participated in this study, with 20 patients enrolled in the Duloxetine and 25 patients in the Nortriptyline group. The mean age of the patients was 37.18 ± 10.62 years, with 27 (60%) females. Demographic analysis showed no significant differences between the two groups regarding age (*p* = 0.221) and sex (*p* = 0.221). Additional demographic findings are presented in Table 1.

**Table 1:** Summary of patient findings during the course of the study.

Characteristics	Time	Intervention group		
		Nortriptyline (*n* = 25)	Duloxetine (*n* = 20)	*p*-value^1^
GSRS	Pre-intervention	31.80 ± 7.14	31.80 ± 6.87	0.909
	First month	30.96 ± 6.97	30.80 ± 7.15	0.940
	Third month	22.04 ± 5.31	25.90 ± 6.26	0.031^*^
	*p*-value^2^	<0.001	<0.001	–
HAS	Pre-intervention	23 (15, 29.50)	22 (11.75, 38.50)	0.504
	First month	22 (15, 25.50)	22 (12.50, 25.75)	0.781
	Third month	19 (14.50, 23)	13 (5.25, 20)	0.049^*^
	*p*-value^2^	<0.001	<0.001	–
HDS	Pre-intervention	14 (8, 34)	12.50 (6.50, 26.75)	0.891
	First month	12 (8.50, 34)	12 (7, 27.25)	0.715
	Third month	12 (8.50, 33.50)	6.50 (2, 17.75)	0.045^*^
	*p*-value^2^	<0.001	<0.001	–
SF-NDI	Pre-intervention	29 (23.50, 34)	29.15 (25, 32.75)	0.896
	First month	19 (12.50, 26)	17.50 (11.25, 23)	0.337
	Third month	15 (9.50, 22.50)	12.50 (7.25, 16.50)	0.046^*^
	*p*-value^2^	<0.001	<0.001	–

^1^Independent samples *t*-test; Mann–Whitney *U* test.

^2^Repeated measure ANOVA, Friedman’s test.

*p < 0.05.

Quantitative data are presented as Mean ± SD; Median (IQR).

GSRS, Gastrointestinal Symptom Rating Scale; HAS, Hamilton Anxiety Score; HDS, Hamilton Depression Score; SF-NDI, Short-form Nepean Dyspepsia Index.

### Temporal changes in symptom severity, anxiety, depression, and quality of life

#### Symptom severity score

Before the study, the mean GSRS score for patient symptom severity was 31.80 ± 6.95, with 31.80 ± 7.14 in the Nortriptyline group and 31.80 ± 6.87 in the Duloxetine group. After one month of intervention, the mean scores were 30.89 ± 6.97, with 30.96 ± 6.97 in the Nortriptyline and 30.80 ± 7.15 in the Duloxetine group. At the third month evaluation, the total score was 23.76 ± 6.01, with 22.04 ± 5.31 in the Nortriptyline and 25.90 ± 6.26 in the Duloxetine group. Statistical analysis revealed no significant difference between the two groups prior to the study (*p* = 0.909) and after the first month of the intervention (*p* = 0.940); however, the severity score was significantly lower in the Nortriptyline group (*p* = 0.031). Details are provided in Table 1 and Supplementary Table S2.

**Figure 2:**
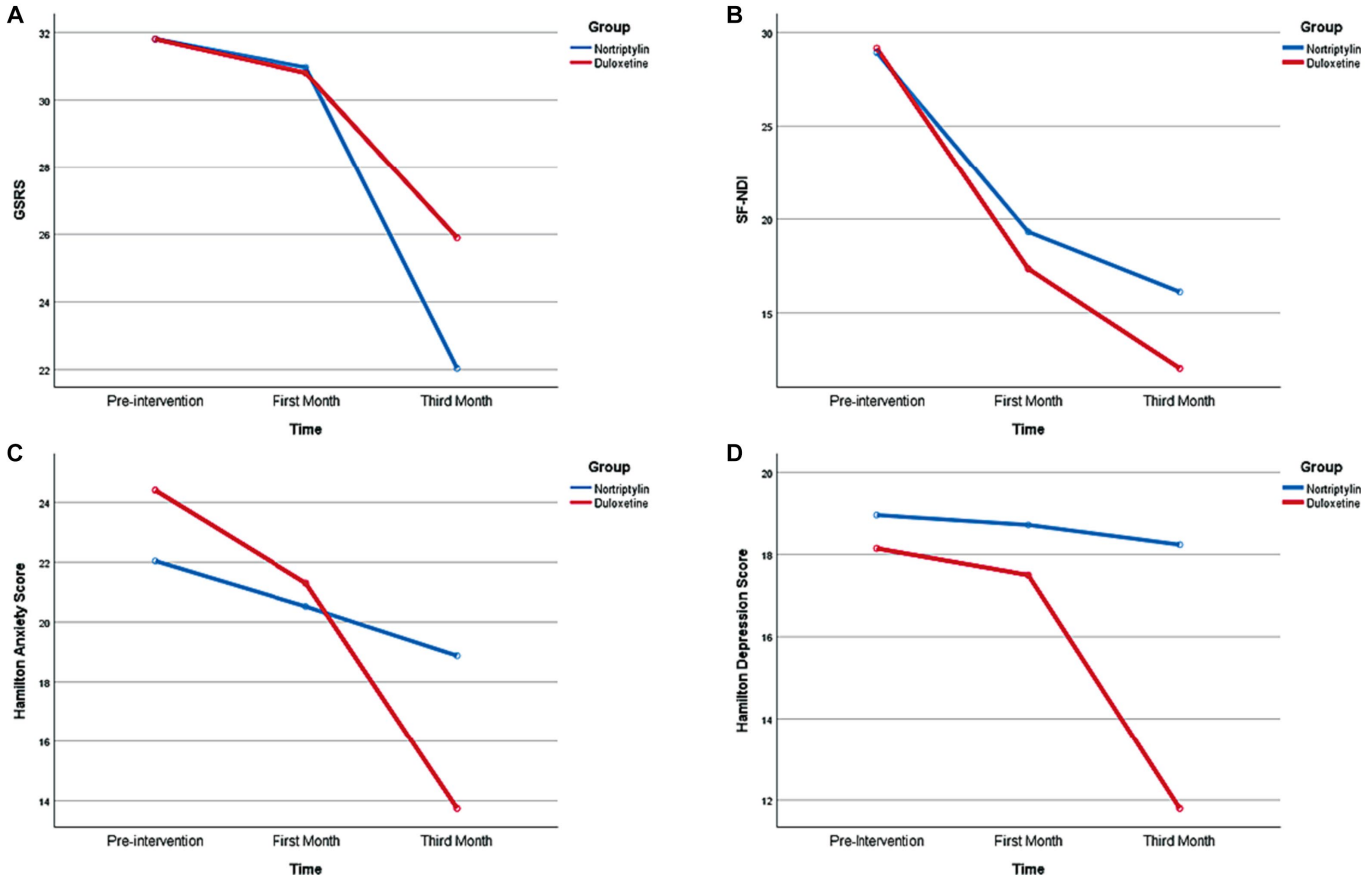
Temporal Changes regarding Symptoms Severity, Anxiety, Depression, and Quality of Life during the time of the Study. GSRS, Gastrointestinal Symptom Rating Scale; SF-NDI, Short-Form Nepean Dyspepsia Index.

Follow-up evaluations in each group showed significant score reductions in both groups in the third month compared to the first month and pre-intervention period. However, no significant score reduction was seen within the first month of the study. Score changes are provided in more detail in Figure 2 and Table 2.

**Table 2:** The impact of Time, Group, their interaction [Group × Time] on the characteristics scores.

Dependent variable	Parameter	B (95% CI)	*p*-value
GSRS	Group [Nortriptyline vs. Duloxetine]	2.07 (−0.81, 4.94)	0.158
	Time	−3.05 (−4.42, −1.68)	<0.001
	Group [Nortriptyline vs. Duloxetine] × Time	−3.27 (−5.14, −1.40)	<0.001
HAS	Group [Nortriptyline vs. Duloxetine]	−2.92 (−7.23, 1.39)	0.184
	Time	−5.15 (−6.67, −3.63)	<0.001
	Group [Nortriptyline vs. Duloxetine] × Time	3.49 (0.86, 6.12)	0.009
HDS	Group [Nortriptyline vs. Duloxetine]	−0.46 (−5.34, 4.42)	0.854
	Time	−3.23 (−5.09, −1.36)	<0.001
	Group [Nortriptyline vs. Duloxetine] × Time	3.07 (0.43, 5.70)	0.023
SF-NDI	Group [Nortriptyline vs. Duloxetine]	0.08 (−3.64, 3.80)	0.965
	Time	−8.25 (−10.09, −6.41)	<0.001
	Group [Nortriptyline vs. Duloxetine] × Time	1.77 (−0.80, 4.34)	0.176

The generalized estimation equation was used to evaluate the impact of Group [Nortriptyline vs. Duloxetine] on the GSRS, HAS, HDS, and SD-NDI over time. GSRS, Gastrointestinal Symptom Rating Scale; HAS, Hamilton Anxiety Score; HDS, Hamilton Depression Score; SF-NDI, Short-form Nepean Dyspepsia Index.

### Hamilton scale

#### Anxiety

Preintervention anxiety assessment revealed a mean anxiety score of 23.09 ± 11.61, with Nortriptyline and Duloxetine group having a mean score of 22.04 ± 9.82 and 24.40 ± 13.68, respectively. One month into the study, the mean anxiety score was 20.87 ± 9.21 in total, 20.52 ± 8.59 in the Nortriptyline, and 21.30 ± 10.15 in the Duloxetine group. The third-month evaluation revealed a total mean score of 16.60 ± 8.73, as Nortriptyline and Duloxetine had a mean of 18.88 ± 8.25 and 13.75 ± 8.68, respectively. Hamilton’s anxiety score demonstrated no significant difference between the two groups in the preintervention (*p* = 0.504) and first-month (*p* = 0.781) assessment, while third-month (*p* = 0.049) evaluation revealed significantly lower scores in the Duloxetine group. Supplementary Table S3 reveals more comprehensive data. However, evaluation of anxiety on a categorical level revealed no significant difference between the two groups at any time points demonstrated in Supplementary Table S4.

Follow-up evaluation in each group revealed a significant score reduction in both groups in the third month compared to the first month and pre-intervention period. However, no significant score reduction was seen within the first month of the study. Score changes are provided in more detail in Figure 2 and Table 2.

### Depression

Preintervention depression assessment revealed a mean anxiety score of 18.60 ± 14.38, with Nortriptyline and Duloxetine group having a mean score of 18.96 ± 14.31 and 18.15 ± 14.84, respectively. One month into the study, the mean depression score was 18.18 ± 13.91 in total and, 18.72 ± 14.07 in the Nortriptyline, and 17.50 ± 14.05 in the Duloxetine group. The third-month evaluation revealed a total mean score of 15.38 ± 13.14, as Nortriptyline and Duloxetine had a mean of 18.24 ± 13.48 and 11.80 ± 12.09, respectively. Hamilton depression score demonstrated no significant difference between the two groups in the preintervention (*p* = 0.891) and first month (*p* = 0.715) assessment, while third month (*p* = 0.045) evaluation revealed significantly lower scores in the Duloxetine group. Supplementary Table S5 provides additional data regarding the depression scores. Evaluation of depression on a categorical level revealed no significant difference between the two groups at any time points presented in Supplementary Table S6.

Follow-up evaluation in each group revealed a significant score reduction in the Duloxetine group in the third month compared to the first month and pre-intervention period. However, no significant score reduction was seen within the first month of the study. Score changes are provided in more detail in Figure 2 and Table 2.

### Quality of life

The mean Nepean score for patient quality of life (SF-NDI), prior to conduction of the study, was 29.02 ± 5.77 with 28.92 ± 6.06 in the Nortriptyline group and 29.15 ± 5.53 in the Duloxetine group. In the first month of the intervention, the mean score was 18.44 ± 6.98, with 19.32 ± 7.13 in the Nortriptyline and 17.35 ± 6.81 in the Duloxetine group. The third-month evaluation revealed a total score of 14.31 ± 6.83, with 16.12 ± 7.17 in the Nortriptyline and 12.05 ± 5.79 in the Duloxetine group. Statistical analysis revealed no significant difference between the two groups prior to the study (*p* = 0.896) and in the first month of the intervention (*p* = 0.337); however, the severity score was significantly lower in the Duloxetine group (*p* = 0.046). Details are provided in Table 1 and Supplementary Table S7.

Follow-up evaluation in each group revealed a significant score reduction in both groups in the third month compared to the first month and pre-intervention period and within the first month of the study. Score changes are provided in more detail in Figure 2 and Table 2.

### Impact of nortriptyline and duloxetine on symptom severity, anxiety, depression, and quality of life over time: insights from generalized estimating equations analysis

Table 2 presents the results of a GEE, evaluating the impact of Group [Nortriptyline vs. Duloxetine] on the GSRS, HAS, HDS, and SF-NDI over time. The impact of Group (Nortriptyline vs. Duloxetine) on GSRS was statistically significant over time (B: −3.27, 95% CI: −5.14 to −1.40, *p* < 0.001). This suggests a more pronounced reduction in GSRS scores over time for the Nortriptyline group compared to the Duloxetine group. Additionally, the Group effect on HAS exhibited significance over time (B: 3.49, 95% CI: 0.86 to 6.12, *p* = 0.009). This implies that the rate of decrease in HAS scores for Nortriptyline was lower than that observed for Duloxetine, highlighting distinct trajectories between the two groups. Furthermore, the mean HDS demonstrated a more rapid decline among the Duloxetine group compared to Nortriptyline over time (B: 3.07, 95% CI: 0.43 to 5.70, *p* = 0.023). This suggests a differential impact of the two treatments on the trajectory of HDS scores. In contrast, the changes in SF-NDI were not found to be significantly different over time between the two groups (B: 1.77, 95% CI: −0.80 to 4.34, *p* = 0.176).

**Figure 3:**
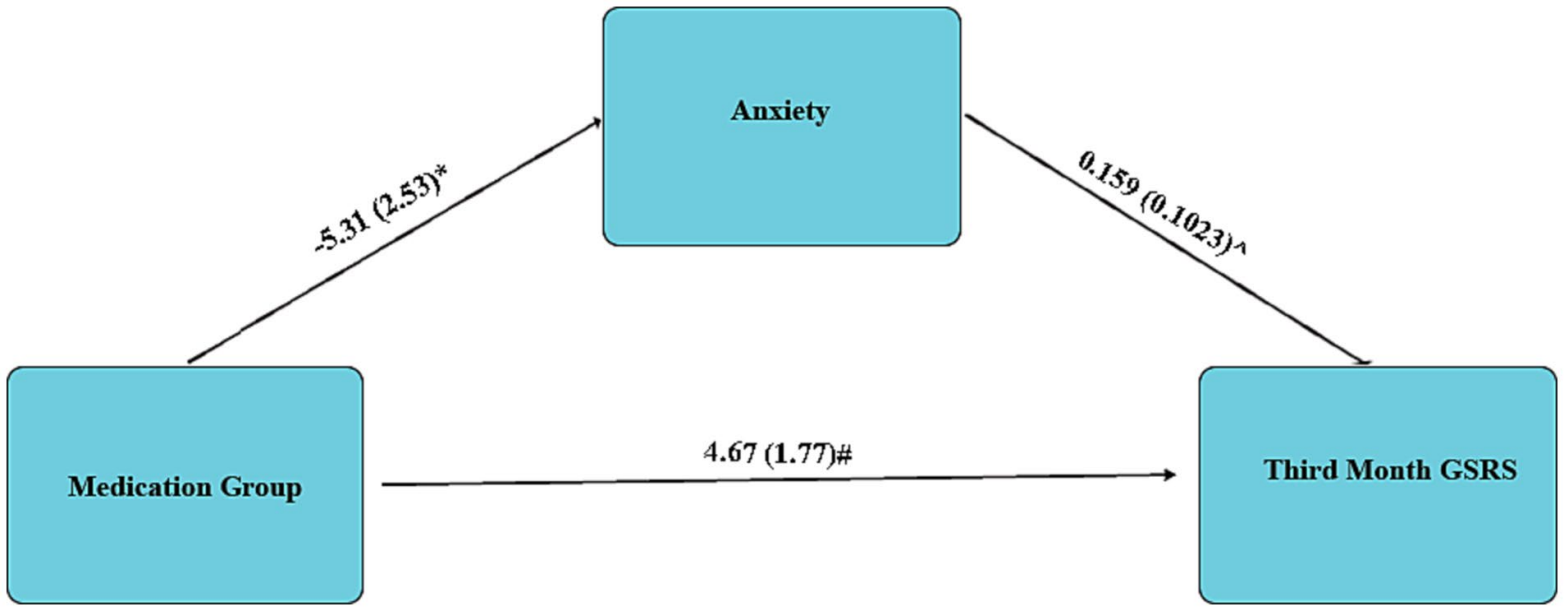
Mediation analysis regarding the mediator role of Anxiety in Third Month GSRS; Data are presented as Coefficient (SE); * 0.049; ^0.0127; # 0.011.

Mediation analysis using linear regression revealed no significant mediation role for depression (Coefficient = −6.44, SE = 3.86, *p* = 0.1031); however, revealed a significant mediator role for anxiety. This mediation analysis revealed a 21.13% reduction for anxiety in the Duloxetine group, presented in the Figure 3.

## Discussion

In this single-blinded randomized clinical trial, our primary objective was to compare the efficacy and safety of Duloxetine and Nortriptyline in the treatment of patients with functional dyspepsia (FD) who had previously shown resistance to Proton Pump Inhibitor (PPI) treatment.

The results of our study revealed that both Duloxetine and Nortriptyline exhibited significant reductions in the severity of FD symptoms by the end of the third month. However, upon closer examination, Nortriptyline demonstrated a statistically significant advantage in reducing symptom severity compared to Duloxetine.

Furthermore, our study investigated the impact of these medications on patients’ psychological well-being. We observed a substantial reduction in anxiety and depression levels in both medication groups by the end of the third month, as compared to baseline and the first month of the study. Notably, Duloxetine significantly alleviated depression in the third month, whereas Nortriptyline did not exhibit a significant improvement in this regard. Additionally, the Duloxetine group displayed significantly lower levels of anxiety and depression than the Nortriptyline group by the end of the third month, although there were no significant differences in anxiety and depression levels between the two groups during the first month.

When assessing the quality of life of patients in both groups, we observed a consistent and significant improvement by the end of the third month compared to baseline and the first month. Significantly, the Duloxetine-treated group reported a substantially better quality of life compared to the Nortriptyline -treated group in the third month. Lastly, we explored the indirect effects of Nortriptyline and Duloxetine on the reduction of FD symptoms through their impact on anxiety and depression levels. Our findings suggest that Duloxetine may have a noteworthy impact, contributing to a 20% reduction in somatic symptoms by lowering anxiety levels.

The use of psychiatric medications is considered a viable treatment option for patients with psychosomatic diagnoses. Studies have investigated the efficacy of TCAs such as Amitriptyline and Nortriptyline in addressing conditions with psychosomatic dimensions, including chronic pain syndrome, Irritable Bowel Syndrome (IBS), and FD (24). Most research has predominantly focused on the role of Amitriptyline in managing IBS, with many studies reporting its positive impact on symptom reduction. However, the extent of this effect varies across studies due to differences in the studied populations and drug dosages (25–28). Conversely, there is a paucity of studies examining the effects of TCAs and other psychiatric drugs on FD. In a study by Jamshidfar et al., which investigated Nortriptyline and Mirtazapine, significant reductions were observed in symptoms such as retrosternal pain, nausea, epigastric pain, and dysphagia in the Nortriptyline -treated group after three months of treatment. Furthermore, depression severity significantly decreased after six weeks and three months of Nortriptyline treatment compared to baseline. Similarly, anxiety levels were significantly lower in patients treated with Nortriptyline after six weeks and three months of treatment compared to pre-treatment levels. However, no significant difference in anxiety levels was observed between the third month and the sixth week of treatment (29). In a systematic review and meta-analysis conducted by Ford et al., improvements in FD symptoms were observed with the use of TCAs and antipsychotics (14). Conversely, Kaosombatwattana et al.’s study, which compared low-dose Nortriptyline to a placebo, did not demonstrate a significant difference in symptom improvement, psychological aspects, or quality of life (30). Additionally, Choung et al.’s study, which investigated low-dose Nortriptyline for two weeks, did not indicate a significant effect on gastric emptying function (31). In our study, the group treated with Nortriptyline exhibited a significant reduction in anxiety and depression levels by the third month compared to baseline. However, despite the decrease in anxiety and depression levels during the first month of treatment, no significant difference was observed in the first month compared to baseline. Similarly, quality of life significantly improved by the third month compared to baseline, with no significant difference noted between the first month and baseline, despite an initial improvement.

Regarding the effect of Duloxetine on FD, no study was found that investigated this issue. However, in the study of Lewis-Fernandez et al., he investigated the effect of Duloxetine in patients with irritable bowel syndrome and major depressive disorder, and in this study, the use of Duloxetine was significantly associated with the improvement of gastrointestinal symptoms, especially abdominal pain (32). Similarly, Brennan et al. delved into the role of Duloxetine in the treatment of IBS, revealing a noteworthy improvement in the quality of life and severity scores related to IBS in individuals administered with Duloxetine (33). In our present study, the use of Duloxetine, akin to Nortriptyline, resulted in significant improvements during the third month compared to baseline and the first month. These improvements encompassed reductions in anxiety and depression levels and enhancements in quality of life.

While psychiatric drugs such as TCAs and SNRIs have shown promise in improving patients’ conditions, the precise mechanisms through which these drugs exert their effects on functional gastrointestinal diseases remain elusive.

Classically, TCAs, including amitriptyline and Nortriptyline, which is a metabolite of amitriptyline (30), are considered as treatment options in digestive functional disorders and show their effects by acting on several levels (29). One proposed mechanism for their efficacy lies in their ability to modulate visceral hypersensitivity. This effect may be attributed, in part, to the non-specific inhibition of serotonin and norepinephrine reabsorption by TCAs within myelinated somatic neuronal pathways (34).

Contemporary research has underscored the significance of the gut-brain axis in the development of various functional gastrointestinal diseases. Several studies suggest that Tricyclic Antidepressants (TCAs) may play a role in alleviating symptoms without directly affecting the digestive system’s function (35). In fact, the activation of a2-adrenergic inhibitory presynaptic receptors in the locus coeruleus by Duloxetine and TCAs can also cause a change in pain perception by reducing the rate of sending sensory messages to the cingulate cortex (33). Likewise, the role of TCA and SNRI drugs in reducing the level of anxiety and depression can also be considered as a factor in reducing the severity of symptoms (36). Additionally, the anti-inflammatory properties of these treatments may also play a role in their therapeutic effects (29).

When side effects restrict the use of TCAs, especially in patients experiencing epigastric pain and displaying intolerance to this class of medications (15), and in cases where gastrointestinal patients concurrently present with depression, SNRIs can serve as a viable alternative (16). SNRIs primarily function by inhibiting the 5-Htand norepinephrine (NA), thereby augmenting the neurotransmission of these substances. Within the SNRI class, there can be variations in the degree of serotonergic and noradrenergic reuptake inhibition among individual drugs. For instance, Venlafaxine predominantly inhibits noradrenaline reuptake, whereas Duloxetine exhibits robust and relatively balanced affinity for both the 5-HT and NA transporters, classifying it as a true SNRI even at lower doses (37). Additionally, SNRIs possess both somatic analgesic and visceral analgesic properties (38).

The present study possesses notable strengths, as It stands as the Inaugural randomized controlled trial assessing the effects of Duloxetine in the context of functional dyspepsia (FD). The three-stage evaluation of patients allows for a comprehensive understanding of the progression of improvement over time. Furthermore, the inclusion of a patient population with no prior history of known psychiatric illness and no prior exposure to psychiatric medications constitutes a noteworthy positive aspect of this study. However, there are certain limitations that warrant consideration. Firstly, the study was conducted within a single center, which may impact the generalizability of the findings. Additionally, the study sample size was relatively small, potentially limiting the statistical power and generalizability of the results.

In conclusion, our study provides valuable insights into the comparative effectiveness of Duloxetine and Nortriptyline in treating FD patients resistant to PPI therapy. While both medications demonstrated efficacy, Nortriptyline appeared to be superior in symptom reduction. Moreover, Duloxetine exhibited more advantages compared to Nortriptyline in addressing anxiety and depression and enhancing the overall quality of life. Further research is warranted to explore the underlying mechanisms driving these differential outcomes and to optimize treatment strategies for patients with FD.

## Data availability statement

The original contributions presented in the study are included in the article/Supplementary material, further inquiries can be directed to the corresponding authors.

## Ethics statement

The studies involving humans were approved by research Ethics Committees of School of Medicine, Shahid Beheshti University of Medical Sciences. The studies were conducted in accordance with the local legislation and institutional requirements. The participants provided their written informed consent to participate in this study.

## Author contributions

SB: Supervision, Conceptualization, Project administration, Writing – review & editing. SM: Project administration, Methodology, Resources, Validation, Writing – original draft. RR: Data curation, Formal analysis, Software, Writing – original draft. AS: Methodology, Supervision, Visualization, Writing – review & editing.

## References

[1] Ford AC Mahadeva S Carbone MF Lacy BE Talley NJ. Functional dyspepsia. Lancet. (2020) 396:1689–702. doi: 10.1016/S0140-6736(20)30469-4 33049222

[2] Ford AC Marwaha A Sood R Moayyedi P. Global prevalence of, and risk factors for, uninvestigated dyspepsia: a meta-analysis. Gut. (2015) 64:1049–57. doi: 10.1136/gutjnl-2014-307843, PMID: 25147201

[3] Black CJ Paine PA Agrawal A Aziz I Eugenicos MP Houghton LA. British Society of Gastroenterology guidelines on the management of functional dyspepsia. Gut. (2022) 71:1697–723. doi: 10.1136/gutjnl-2022-327737, PMID: 35798375 PMC9380508

[4] Yamawaki H Futagami S Wakabayashi M Sakasegawa N Agawa S Higuchi K. Management of functional dyspepsia: state of the art and emerging therapies. Ther Adv Chronic Dis. (2018) 9:23–32. doi: 10.1177/2040622317725479, PMID: 29344328 PMC5761940

[5] Clauwaert N Jones M Holvoet L Vandenberghe J Vos R Tack J. Associations between gastric sensorimotor function, depression, somatization, and symptom-based subgroups in functional gastroduodenal disorders: are all symptoms equal? Neurogastroenterol Motil. (2012) 24:1088–e565. doi: 10.1111/j.1365-2982.2012.01985.x 22816492

[6] Nan J Liu J Mu J Dun W Zhang M Gong Q. Brain-based correlations between psychological factors and functional dyspepsia. J Neurogastroenterol Motil. (2015) 21:103–10. doi: 10.5056/jnm14096, PMID: 25540947 PMC4288085

[7] Van Oudenhove L Aziz Q. The role of psychosocial factors and psychiatric disorders in functional dyspepsia. Nat Rev Gastroenterol Hepatol. (2013) 10:158–67. doi: 10.1038/nrgastro.2013.10, PMID: 23358396

[8] Van Oudenhove L Vandenberghe J Vos R Holvoet L Demyttenaere K Tack J. Risk factors for impaired health-related quality of life in functional dyspepsia. Aliment Pharmacol Ther. (2011) 33:261–74. doi: 10.1111/j.1365-2036.2010.04510.x, PMID: 21083672

[9] Sclocco R Fisher H Staley R Han K Mendez A Bolender A. Cine gastricMRIreveals alteredGut–BrainAxis in functional dyspepsia: gastric motility is linked with brainstem‐corticalfMRIconnectivity. Neurogastroenterol Motil. (2022) 34:e14396. doi: 10.1111/nmo.14396, PMID: 35560690 PMC9529794

[10] Talley NJ Ford AC. Functional dyspepsia. N Engl J Med. (2015) 373:1853–63. doi: 10.1056/NEJMra1501505 26535514

[11] Agirman G Yu KB Hsiao EY. Signaling inflammation across the gut-brain axis. Science. (2021) 374:1087–92. doi: 10.1126/science.abi6087, PMID: 34822299

[12] Mayer EA Nance K Chen S. The gut–brain axis. Annu Rev Med. (2022) 73:439–53. doi: 10.1146/annurev-med-042320-014032 34669431

[13] Zhou W Li X Huang Y Xu X Liu Y Wang J. Comparative efficacy and acceptability of psychotropic drugs for functional dyspepsia in adults: a systematic review and network meta-analysis. Medicine. (2021) 100:e26046. doi: 10.1097/MD.0000000000026046 34011118 PMC8137050

[14] Ford AC Luthra P Tack J Boeckxstaens GE Moayyedi P Talley NJ. Efficacy of psychotropic drugs in functional dyspepsia: systematic review and meta-analysis. Gut. (2017) 66:411–20. doi: 10.1136/gutjnl-2015-310721, PMID: 26567029

[15] Drossman DA Tack J Ford AC Szigethy E Törnblom H Van Oudenhove L. Neuromodulators for functional gastrointestinal disorders (disorders of gut− brain interaction): a Rome foundation working team report. Gastroenterology. (2018) 154:1140–1171.e1. doi: 10.1053/j.gastro.2017.11.279, PMID: 29274869

[16] Törnblom H Drossman DA. Psychotropics, antidepressants, and visceral analgesics in functional gastrointestinal disorders. Curr Gastroenterol Rep. (2018) 20:1–10. doi: 10.1007/s11894-018-0664-3 30397821 PMC6223713

[17] Revicki DA Wood M Wiklund I Crawley J. Reliability and validity of the gastrointestinal symptom rating scale in patients with gastroesophageal reflux disease. Qual Life Res. (1997) 7:75–83. doi: 10.1023/A:1008841022998 9481153

[18] Mazaheri M SadatKhoshouei M. Comparison between psychometric characteristics of Persian version of the gastrointestinal symptoms rating scale in functional gastrointestinal disorders and normal groups. Govaresh. (2012) 17:18–24.

[19] Hamilton M. Hamilton anxiety scale. Group. (1959) 1:10–1037.

[20] Hamilton M. The Hamilton depression scale—accelerator or break on antidepressant drug discovery. Psychiatry. (1960) 23:56–62. 10.1136/jnnp-2013-306984 24443712

[21] Trajković G Starčević V Latas M Leštarević M Ille T Bukumirić Z. Reliability of the Hamilton rating scale for depression: a meta-analysis over a period of 49 years. Psychiatry Res. (2011) 189:1–9. doi: 10.1016/j.psychres.2010.12.007, PMID: 21276619

[22] Ahmadpanah M Sheikhbabaei M Haghighi M Roham F Jahangard L Akhondi A. Validity and test–retest reliability of the Persian version of the Montgomery–asberg depression rating scale. Neuropsychiatr Dis Treat. (2016) 12:603–7. doi: 10.2147/NDT.S103869 27022265 PMC4788359

[23] Talley NJ Haque M Wyeth JW Stace NH Tytgat GN Stanghellini V. Development of a new dyspepsia impact scale: the Nepean dyspepsia index. Aliment Pharmacol Ther. (1999) 13:225–35. doi: 10.1046/j.1365-2036.1999.00445.x, PMID: 10102954

[24] Lacy B Talley N Locke G III Bouras E DiBaise J El-Serag H. Current treatment options and management of functional dyspepsia. Aliment Pharmacol Ther. (2012) 36:3–15. doi: 10.1111/j.1365-2036.2012.05128.x, PMID: 22591037 PMC3970847

[25] Alderson SL Wright-Hughes A Ford AC Farrin A Hartley S Fernandez C. Amitriptyline at low-dose and titrated for irritable bowel syndrome as second-line treatment (the ATLANTIS trial): protocol for a randomised double-blind placebo-controlled trial in primary care. Trials. (2022) 23:1–12. doi: 10.1186/s13063-022-06492-6 35804433 PMC9264306

[26] Chao G-q Zhang S. A meta-analysis of the therapeutic effects of amitriptyline for treating irritable bowel syndrome. Intern Med. (2013) 52:419–24. doi: 10.2169/internalmedicine.52.9147 23411695

[27] Ford AC Wright-Hughes A Alderson S Ow PL Ridd M Foy R. O51 amitriptyline at low-dose and titrated for irritable bowel syndrome as second-line treatment (the ATLANTIS study): a double-blind placebo-controlled trial BMJ Publishing Group (2023).

[28] Rajagopalan M Rajagopalan A Rajagopalan A. Amitriptyline in irritable bowel syndrome: a clinical review. Curr Med Issues. (2021) 19:110–4. doi: 10.4103/cmi.cmi_13_21

[29] Jamshidfar N Hamdieh M Eslami P Batebi S Sadeghi A Rastegar R. Comparison of the potency of nortriptyline and mirtazapine on gastrointestinal symptoms, the level of anxiety and depression in patients with functional dyspepsia. Gastroenterol Hepatol Bed Bench. (2023) 16:468–77. doi: 10.22037/ghfbb.v16i1.2513 37070114 PMC10105507

[30] Kaosombatwattana U Pongprasobchai S Limsrivilai J Maneerattanaporn M Leelakusolvong S Tanwandee T. Efficacy and safety of nortriptyline in functional dyspepsia in Asians: a randomized double-blind placebo-controlled trial. J Gastroenterol Hepatol. (2018) 33:411–7. doi: 10.1111/jgh.13914 28768370

[31] Choung RS Cremonini F Thapa P Zinsmeister AR Talley N. The effect of short-term, low-dose tricyclic and tetracyclic antidepressant treatment on satiation, postnutrient load gastrointestinal symptoms and gastric emptying: a double-blind, randomized, placebo-controlled trial. Neurogastroenterol Motil. (2008) 20:220–7. doi: 10.1111/j.1365-2982.2007.01029.x, PMID: 18031471

[32] Lewis-Fernández R Lam P Lucak S Galfalvy H Jackson E Fried J. An open-label pilot study of duloxetine in patients with irritable bowel syndrome and comorbid major depressive disorder. J Clin Psychopharmacol. (2016) 36:710–5. doi: 10.1097/JCP.0000000000000599, PMID: 27755218

[33] Brennan BP Fogarty KV Roberts JL Reynolds KA Pope HG Jr Hudson JI. Duloxetine in the treatment of irritable bowel syndrome: an open-label pilot study. Hum Psychopharmacol Clin Exp. (2009) 24:423–8. doi: 10.1002/hup.1038, PMID: 19548294

[34] Prakash C Lustman PJ Freedland KE Clouse RE. Tricyclic antidepressants for functional nausea and vomiting (clinical outcome in 37 patients). Dig Dis Sci. (1998) 43:1951–6. doi: 10.1023/A:1018878324327 9753257

[35] Masuy I Van Oudenhove L Tack J. Treatment options for functional dyspepsia. Aliment Pharmacol Ther. (2019) 49:1134–72. doi: 10.1111/apt.15191 30924176

[36] Thiwan SI Drossman DA. Treatment of functional GI disorders with psychotropic medicines: a review of evidence with a practical approach. Gastroenterol Hepatol. (2006) 2:678–88. PMID: 28316538 PMC5350580

[37] Stahl SM. Essential psychopharmacology: Neuroscientific basis and practical applications. Cambridge: Cambridge university press (2000).

[38] Johnson AC Greenwood-Van Meerveld B. The pharmacology of visceral pain. Adv Pharmacol. (2016) 75:273–301. doi: 10.1016/bs.apha.2015.11.002 26920016

