# Supplementary material for: Comparing the efficacy of duloxetine and nortriptyline in alleviating the symptoms of functional dyspepsia – a randomized clinical trial
Source: Front Psychiatry. 2024 Jan 16;14:1297231. doi: 10.3389/fpsyt.2023.1297231 (PMC10824943; doi:10.3389/fpsyt.2023.1297231)
Supplement: Supplementary file 1 [file Table_1.DOCX]

**Supplementary data**

Table S1. Age distribution between nortriptyline and duloxetine groups

Table S2. Assessing sex distribution between the two groups

Table S3. Severity Score distribution between the two groups at each time point

Table S4. Hamilton Anxiety Score distribution between the two groups at each time point

Table S5. Hamilton Anxiety categories between the two groups at each time point

Table S6. Hamilton Depression Score distribution between the two groups at each time point

Table S7. Hamilton Depression categories between the two groups at each time point

Table S8. Short-form Nepean Dyspepsia Index (SF-NDI) between the two groups at each time-point

Table S9. Temporal changes regarding symptom severity, anxiety, depression, and quality of life

| Table S1. Assessing Age distribution between the two groups | | | | | | |
| --- | --- | --- | --- | --- | --- | --- |
|  | **Groups** | **Mean± SD** | **Median** | **Min, Max** | **IQR** | **P-value^1^** |
| Age | **Total** | 37.18± 10.62 | 38.00 | 20, 55 | 28.00-45.00 | 0.221 |
|  | **Nortriptyline** | 35.52±11.36 | 34.00 | 20, 55 | 25.00-47.00 |  |
|  | **Duloxetine** | 39.25±9.48 | 41.00 | 23, 53 | 31.25-45.75 |  |
| ^1^: Independent Samples T-test | | | | | | |

| Table S2. Assessing sex distribution between the two groups | | | |
| --- | --- | --- | --- |
|  | **Sex** | |  |
| Group | **Male** | **Female** | **P-value^1^** |
| Total | 18 (40) | 27 (60) | 0.221 |
| Nortriptyline | 12 (48) | 13 (52) |  |
| Duloxetine | 6 (30) | 14 (70) |  |
| Qualitative data are presented as count (percentage)  ^1^: Chi-Square test | | | |

| Table S3. Assessing the Severity Score distribution between the two groups | | | | | | |
| --- | --- | --- | --- | --- | --- | --- |
| Time | **Groups** | **Mean± SD** | **Median** | **Min, Max** | **IQR** | **P-value^1^** |
| Pretest | Total | 31.80±6.95 | 33.00 | 21, 42 | 25.00-38.00 | 0.909 |
|  | Nortriptyline | 31.80±7.14 | 33.00 | 21,42 | 25.00-38.50 |  |
|  | Duloxetine | 31.80±6.87 | 32.50 | 21,41 | 24.50-38.00 |  |
| First Month | Total | 30.89±6.97 | 31.00 | 19,45 | 25.00-37.00 | 0.940 |
|  | Nortriptyline | 30.96±6.97 | 30.00 | 21,45 | 24.50-37.00 |  |
|  | Duloxetine | 30.80±7.15 | 31.50 | 19,42 | 25.00-37.00 |  |
| Third Month | Total | 23.76±6.01 | 25.00 | 13, 37 | 18.00-28.00 | 0.031 |
|  | Nortriptyline | 22.04±5.31 | 21.00 | 13,31 | 17.00-26.00 |  |
|  | Duloxetine | 25.90±6.26 | 27.50 | 17,37 | 19.00-31.00 |  |
| ^1^: Independent Samples T-test; Mann-Whitney U test | | | | | | |

| Table S4. Assessing the Hamilton Anxiety Score between the two groups | | | | | | |
| --- | --- | --- | --- | --- | --- | --- |
| Time | **Groups** | **Mean± SD** | **Median** | **Min, Max** | **IQR** | **P-value^1^** |
| Pretest | Total | 23.09± 11.61 | 22 | 3, 47 | 14.00-30.50 | 0.504 |
|  | Nortriptyline | 22.04± 9.82 | 23 | 3, 44 | 15-29.50 |  |
|  | Duloxetine | 24.40± 13.68 | 22 | 7, 47 | 11.75-38.50 |  |
| First Month | Total | 20.87± 9.21 | 22 | 4, 43 | 14.00-25.50 | 0.781 |
|  | Nortriptyline | 20.52±8.59 | 22 | 4, 43 | 15.00-25.50 |  |
|  | Duloxetine | 21.30± 10.15 | 22 | 7, 40 | 12.50-25.75 |  |
| Third Month | Total | 16.60±8.73 | 16 | 3, 43 | 10.00-22.00 | 0.049 |
|  | Nortriptyline | 18.88±8.25 | 19 | 4, 43 | 14.50-23.00 |  |
|  | Duloxetine | 13.75±8.68 | 13 | 3, 31 | 5.25-20.00 |  |
| ^1^: Independent Samples T-test; Mann-Whitney U test | | | | | | |

| Table S5. Assessing the Hamilton Anxiety categories between the two groups | | | | | |
| --- | --- | --- | --- | --- | --- |
| Time | **Levels** | **Groups** | | | **P-value^1^** |
|  |  | **Total** | **Nortriptyline** | **Duloxetine** |  |
| Pretest | Mild (<17) | 14 (31.1) | 7 (28) | 7 (35) | 0.715 |
|  | Mild to Moderate (18-24) | 14 (31.1) | 9 (36) | 5 (25) |  |
|  | Moderate to Severe (25-30) | 6 (13.3) | 4 (16) | 2 (10) |  |
|  | Severe (>30) | 11 (24.4) | 5 (20) | 6 (30) |  |
| First Month | Mild (<17) | 16 (35.6) | 9 (36) | 7 (35) | 0.609 |
|  | Mild to Moderate (18-24) | 16 (35.6) | 10 (40) | 6 (30) |  |
|  | Moderate to Severe (25-30) | 9 (20) | 5 (20) | 4 (20) |  |
|  | Severe (>30) | 4 (8.9) | 1 (4) | 3 (15) |  |
| Third Month | Mild (<17) | 25 (55.6) | 10 (40) | 15 (75) | 0.073 |
|  | Mild to Moderate (18-24) | 13 (28.9) | 11 (44) | 2 (10) |  |
|  | Moderate to Severe (25-30) | 5 (11.1) | 3 (12) | 2 (10) |  |
|  | Severe (>30) | 2 (4.4) | 1 (4) | 1 (5) |  |
| Data is presented as n (%)  ^1^: Chi-Square Test | | | | | |

| Table S6. Assessing the Hamilton Depression Score between the two groups | | | | | | |
| --- | --- | --- | --- | --- | --- | --- |
| Time | **Groups** | **Mean± SD** | **Median** | **Min, Max** | **IQR** | **P-value^1^** |
| Pretest | Total | 18.60± 14.38 | 14 | 1, 47 | 8.00-31 | 0.891 |
|  | Nortriptyline | 18.96±14.31 | 14 | 1, 43 | 8.00-34.00 |  |
|  | Duloxetine | 18.15± 14.84 | 12.50 | 1, 47 | 6.50-26.75 |  |
| First Month | Total | 18.18± 13.91 | 12 | 1, 44 | 7.00-31 | 0.715 |
|  | Nortriptyline | 18.72±14.07 | 12 | 2, 43 | 8.50-34.00 |  |
|  | Duloxetine | 17.50± 14.05 | 12 | 1, 44 | 7.00-27.25 |  |
| Third Month | Total | 15.38± 13.14 | 11 | 1, 41 | 3.50-28.00 | 0.045 |
|  | Nortriptyline | 18.24±13.48 | 12 | 2, 41 | 8.50-33.50 |  |
|  | Duloxetine | 11.80± 12.09 | 6.50 | 1, 37 | 2.00-17.75 |  |
| ^1^: Independent Samples T-test; Mann-Whitney U test | | | | | | |

| Table S7. Assessing the Hamilton Depression categories between the two groups | | | | | |
| --- | --- | --- | --- | --- | --- |
| Time | **Levels** | **Groups** | | | **P-value^1^** |
|  |  | **Total** | **Nortriptyline** | **Duloxetine** |  |
| Pretest | Absence or Remission (<7) | 10 (22.2) | 5 (20) | 5 (25) | 0.967 |
|  | Mild (7-17) | 17 (37.8) | 10 (40) | 7 (35) |  |
|  | Moderate (18-24) | 4 (8.9) | 2 (8) | 2 (10) |  |
|  | Severe (>24) | 14 (31.3) | 8 (32) | 6 (30) |  |
| First Month | Absence or Remission (<7) | 9 (20) | 5 (20) | 4 (20) | 0.981 |
|  | Mild (7-17) | 18 (40) | 10 (40) | 8 (40) |  |
|  | Moderate (18-24) | 3 (6.7) | 2 (8) | 1 (5) |  |
|  | Severe (>24) | 15 (33.3) | 8 (32) | 7 (35) |  |
| Third Month | Absence or Remission (<7) | 14 (31.1) | 4 (16) | 10 (50) | 0.111 |
|  | Mild (7-17) | 16 (35.6) | 11 (44) | 5 (25) |  |
|  | Moderate (18-24) | 3 (6.7) | 2 (8) | 1 (5) |  |
|  | Severe (>24) | 12 (26.7) | 8 (32) | 4 (20) |  |
| Data is presented as n (%)  ^1^: Chi-Square test | | | | | |

| Table S8. Assessing the SF-NDI score between the two groups | | | | | | |
| --- | --- | --- | --- | --- | --- | --- |
| Time | **Groups** | **Mean± SD** | **Median** | **Min, Max** | **IQR** | **P-value^1^** |
| Pretest | **Total** | 29.02±5.77 | 29 | 18, 41 | 24.50-33.50 | 0.896 |
|  | **Nortriptyline** | 28.92±6.06 | 29 | 18, 38 | 23.50-34.00 |  |
|  | **Duloxetine** | 29.15±5.53 | 29.15 | 21, 41 | 25.00-32.75 |  |
| First Month | **Total** | 18.44±6.98 | 19 | 7,31 | 12.00-24.50 | 0.337 |
|  | **Nortriptyline** | 19.32±7.13 | 19 | 9, 31 | 12.50-26.00 |  |
|  | **Duloxetine** | 17.35±6.81 | 17.50 | 7, 28 | 11.25-23.00 |  |
| Third Month | **Total** | 14.31±6.83 | 14 | 3, 29 | 8.50- 19.5 | 0.046 |
|  | **Nortriptyline** | 16.12±7.17 | 15 | 6, 29 | 9.50-22.50 |  |
|  | **Duloxetine** | 12.05±5.79 | 12.50 | 3, 23 | 7.25-16.50 |  |
| SF-NDI: Short-form Nepean Dyspepsia Index  ^1^: Independent Samples T-test; Mann-Whitney U test | | | | | | |

| Table S9. Temporal changes regarding symptom severity, anxiety, depression, and quality of life | | | | | |
| --- | --- | --- | --- | --- | --- |
| Characteristics | **Groups** | **Time** | | | **Group × time** |
|  |  | **Pre-Intervention** | **First Month** | **Third Month** | **P-value^1^** |
| GSRS | Nortriptyline | 31.80±7.14 | 30.96±6.97 | 22.04±5.31 ^a,b^ | <0.001 |
|  | Duloxetine | 31.80±6.87 | 30.80±7.15 | 25.90±6.26 ^c,d^ | <0.001 |
| HAS | Nortriptyline | 22.04± 9.82 | 20.52±8.59 | 18.88±8.25 ^e,f^ | <0.001 |
|  | Duloxetine | 24.40± 13.68 | 21.30± 10.15 | 13.75±8.68 ^g,h^ | <0.001 |
| HDS | Nortriptyline | 18.96±14.31 | 18.72±14.07 | 18.24±13.48 ^i^ | <0.001 |
|  | Duloxetine | 18.15± 14.84 | 17.50± 14.05 | 11.80± 12.09 ^j,k^ | <0.001 |
| SF-NDI | Nortriptyline | 28.92±6.06 | 19.32±7.13 ^m^ | 16.12±7.17 ^n,o^ | <0.001 |
|  | Duloxetine | 29.15±5.53 | 17.35±6.81 ^p^ | 12.05±5.79 ^q,r^ | <0.001 |
| GSRS: Gastrointestinal Symptom Rating Scale; HAS: Hamilton Anxiety Score; HDS: Hamilton Depression Score; SF-NDI: Short-form Nepean Dyspepsia Index  ^1^: Repeated Measure ANOVA test  ^a^: p-value regarding third-month and pre-intervention changes: p<0.001  ^b^: p-value regarding third-month and first-month changes: p<0.001  ^c^: p-value regarding third-month and pre-intervention changes: p<0.001  ^d^: p-value regarding third-month and first-month changes: p<0.001  ^e^: p-value regarding third-month and pre-intervention changes: p=0.003  ^f^: p-value regarding third-month and first-month changes: p<0.001  ^g^: p-value regarding third-month and pre-intervention changes: p<0.001  ^h^: p-value regarding third-month and first-month changes: p<0.001  ^i^: p-value regarding Friedman\s test: p=0.081  ^j^: p-value regarding third-month and pre-intervention changes: p<0.001  ^k^: p-value regarding third-month and first-month changes: p<0.001  ^m^: p-value regarding first-month and pre-intervention changes: p<0.001  ^n^: p-value regarding third-month and pre-intervention changes: p<0.001  ^o^: p-value regarding third-month and first-month changes: p<0.001  ^p^: p-value regarding first-month and pre-intervention changes: p<0.001  ^q^: p-value regarding third-month and pre-intervention changes: p<0.001  ^r^: p-value regarding third-month and first-month changes: p<0.001 | | | | | |
